# Bibliography

**Published:** 1891-12

**Authors:** 


### Bibliography.

THE PHYSICIAN'S VISITING LIST FOR 1892.

The Physician's Visiting List has just been issued by P. Blakiston, Son & Co., of Philadelphia, Pa.

This is the forty-first year of the publication of this little work. It is a remarkably comprehensive little work; amongst other things it contains a list of new remedies, Incompatibility, Poisons and Antidotes, revised for 1892, Disinfectants, Diagnosis and treatment of the simpler diseases of the Eye, Asphyxia, Apnœa, etc.

The Visiting List is sufficient for the record of twenty-five patients per week. There is also contained in it a great amount of valuable information in a very small space, a large share of which is interesting to the Dentist as well as to the Physician, and because of this every Dentist should have a copy of this work; it can also be used as an engagement book. It can be obtained of the Publishers or through Booksellers.

